# Sex differences in amyloid transthyretin cardiomyopathy National Amyloidosis Centre staging at diagnosis: an analysis from a large single-center cohort

**DOI:** 10.3389/fcvm.2026.1900402

**Published:** 2026-09-21

**Authors:** C. Mutebi, N. Kusi, K. C. Ositelu, O. Oputa, K. Rasberry, P. Cremer, Q. Youmans, S. Lehrer, S. Shah, A. Tibrewala, R. L. Weinberg, I. S. Okwuosa

**Affiliations:** 1 Department of Medicine, Feinberg School of Medicine, Northwestern University, Chicago, IL, United States; 2 Feinberg School of Medicine, Northwestern University, Chicago, IL, United States; 3 Division of Cardiology, Feinberg School of Medicine, Northwestern University, Chicago, IL, United States

**Keywords:** cardiac amyloid, sex differences, survival, transthyretin (ATTR) amyloidosis, TTR

## Abstract

**Background:**

Amyloid transthyretin cardiomyopathy (ATTR-CM) is a progressive infiltrative cardiomyopathy that has seen significant advances in diagnosis and treatment over the last two decades. Sex differences in phenotype, treatment response, and long-term outcomes remain poorly understood. Our study aims to describe the factors that contribute to differences in phenotype and long-term outcomes between male and female patients.

**Methods:**

This was a single-center retrospective analysis of 334 patients with ATTR-CM seen at Northwestern Memorial Hospital, Chicago, IL, USA. The study included adults >18 years with an ATTR-CM diagnosis (wild-type, variant). Immunoglobulin light chain (AL) amyloidosis patients were excluded. Patients were classified as male or female based on the biological gender described in the electronic medical record. Male patients with ATTR-CM were compared with female patients with ATTR-CM. The National Amyloidosis Centre (NAC) stage was calculated using clinical parameters at the time of diagnosis. The primary outcome was all-cause mortality. Baseline characteristics were compared using chi-squared analysis. Associations were determined using Kaplan–Meier estimation and restricted mean survival time analysis (RMST).

**Results:**

The study included 334 patients with ATTR-CM—268 (80%) men and 66 (20%) women. Female patients were diagnosed at higher NAC stages (*p* = 0.002), had lower pre-albumin levels and a lower estimated glomerular filtration rate, higher N-terminal pro-B-type natriuretic peptide levels, a higher prevalence of the ATTR variant genotype, and were more likely to be African American. During a median follow-up of 42 months (IQR 17–70), 74 deaths occurred. The unadjusted 60-month RMST difference between men and women was −2.2 months (*p* = 0.37). In our multivariable analysis, age at diagnosis (*p* < 0.001) and atrial fibrillation (*p* = 0.017) were the only factors associated with increased mortality.

**Conclusions:**

Female patients present with ATTR-CM at significantly later NAC stages than men. Despite this, they have a similar mortality rate. Our findings may demonstrate that the NAC stage overestimates disease severity in female patients or, alternatively, that biological mechanisms confer a less aggressive disease phenotype in female patients despite advanced disease staging.

## Graphical Abstract

**Figure:**
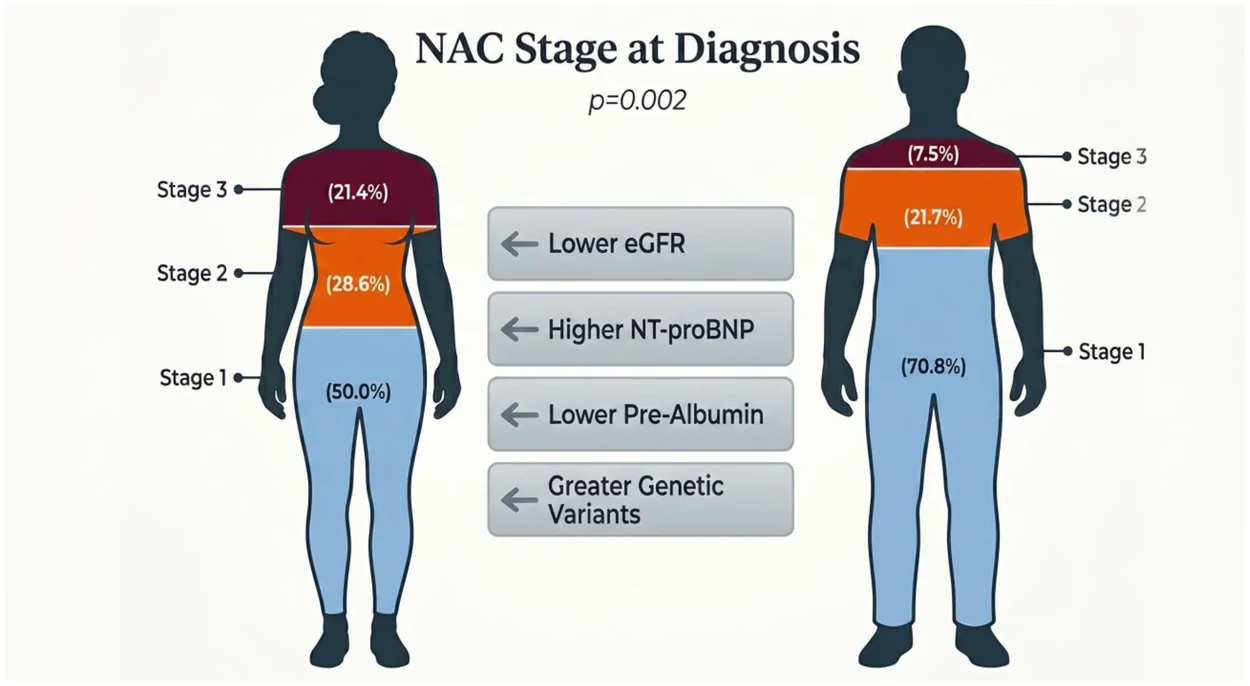

## Highlights

- Female patients with amyloid transthyretin cardiomyopathy present at a higher National Amyloidosis Centre stage than male patients.
- Age at diagnosis and atrial fibrillation were independent predictors of mortality.
- Although presenting at a higher stage of disease, female patients experience similar mortality to male patients.
- Standardized algorithms and referral processes may help diagnose female patients at earlier stages.

## Introduction

Amyloid transthyretin cardiomyopathy (ATTR-CM) is a restrictive cardiomyopathy characterized by extracellular deposition of misfolded transthyretin fibrils in the myocardium (1). Previously regarded as a rare condition, ATTR-CM has undergone a substantial reappraisal over the last two decades. The advent of radionuclide imaging for ATTR-CM has revealed a higher prevalence of the disease (2, 3). In addition, large cohorts of patients with ATTR-CM have helped derive scoring systems such as the National Amyloidosis Centre (NAC) for prognostic staging (4). In parallel with increased recognition, outcomes have improved in recent years, an effect driven by the introduction of transthyretin-stabilizing agents (5). Longitudinal cohort analyses comparing pre- and post-2019 diagnostic eras consistently demonstrate superior survival, a trend attributed to earlier disease identification, more refined phenotypic characterization, and expanded access to disease-modifying therapeutics such as stabilizers and silencer therapies (5, 6). Nonetheless, the longstanding paradigm that ATTR-CM predominantly affects older men continues to influence diagnostic evaluation, referral pathways, and clinical trial enrollment, thereby limiting a comprehensive understanding of disease expression across sexes (7, 8).

Sex-based differences in cardiovascular epidemiology are shaped by interactions among hormonal, genetic, structural, and sociocultural determinants. Recent literature suggests that similar sex-related variation exists in ATTR-CM. Both wild-type (WT-ATTR-CM) and variant (V-ATTR-CM) subtypes are reported to comprise 80%–90% men in multiple cohort studies (1, 9, 10). However, the true burden in women remains insufficiently quantified. Notably, a study employing active case ascertainment found that women accounted for 31.3% of individuals with confirmed ATTR-CM, compared with only 13.3% detected through standard referral pathways, underscoring a substantial and likely systemic detection deficit (11). Further, several analyses have suggested that women may exhibit greater indexed left ventricular wall thickness and a higher disease stage when normalized for height, demonstrating an effect of delayed or underdiagnosis (10, 12). In contrast, two of the largest contemporary investigations evaluating sex differences in ATTR-CM reported no significant differences in stage at diagnosis or subsequent clinical outcomes, including disease progression or mortality (13, 14). Collectively, these findings underscore the conflicting and incompletely understood sex-based differences in disease onset, phenotype, and clinical trajectory in ATTR-CM.

There has been progress in understanding how ATTR-CM presents differently in male and female patients, but considerable questions persist. Risk profiles, disease trajectory patterns, and long-term outcomes remain inadequately defined. This study aims to delineate sex-based differences in the presentation and long-term outcomes of ATTR-CM in a contemporary clinical cohort. In addition, we seek to identify demographic and clinical factors that may contribute to differential disease burden between male and female patients.

## Methods

### Study population

This was a single-center study of patients evaluated at Cardiac Amyloidosis Center of Northwestern Memorial Hospital in Chicago, IL, USA, who were captured in the amyloidosis registry of the institution. The registry includes patients seen at the clinic from 28 May 2010 to 1 June 2024.

### Patient selection

Included in this study were adults aged 18 years or older with a confirmed diagnosis of ATTR-CM at Northwestern Medicine Health System. The diagnosis was confirmed by one of the two methods: (1) endomyocardial biopsy with mass spectroscopy fiber typing or (2) nuclear scintigraphy strongly suggestive of TTR amyloidosis, with serum exclusion of light chains. A nuclear scintigraphy examination with grade 2 or 3 uptake was reported as “Strongly suggestive of ATTR-CM,” in line with guidelines from the American Society of Nuclear Cardiology (15). Individuals with other types of amyloidosis, such as immunoglobulin light chain (AL) amyloidosis and isolated atrial amyloidosis, were excluded from this analysis. Patients were further stratified by sex as self-identified male or female (M or F).

Baseline characteristics, including age at diagnosis, sex at birth, race/ethnicity, biomarkers at the time of diagnosis [pre-albumin, estimated glomerular filtration rate (eGFR), N-terminal pro-B-type natriuretic peptide (NT-proBNP)], NAC stage, history of heart failure, history of hospitalization, history of ventricular tachycardia (VT), and history of non-sustained ventricular tachycardia (NSVT), were collected. The NAC staging system is an internationally recognized system that classifies patients with ATTR-CM into prognostic categories based on NT-proBNP levels and eGFR (stage 1: NT-proBNP ≤3,000 ng/L and eGFR ≥45 mL/min; stage 3: NT-proBNP >3,000 ng/L and eGFR <45 mL/min; stage 2: all other combinations) (4). A fourth stage encompasses individuals with an NT-proBNP >10,000 ng/L (16). Higher stages are independently associated with all-cause mortality, helping to stratify patient risk at the time of diagnosis (4). Individuals were classified as having WT-ATTR-CM or V-ATTR-CM based on TTR genetic testing. Individuals with an unknown genotype or a variant of unknown significance (VUS) were included in the WT-ATTR-CM subgroup.

### Outcomes

The primary outcomes for this analysis were (1a) disease severity at time of diagnosis based on NAC staging and (1b) all-cause mortality. The secondary outcome was all-cause hospitalization. The number of hospitalizations was converted to a dichotomous variable of no hospitalizations vs. any hospitalizations in the first year after diagnosis because of the severe right skew of the data. Because of the smaller number of events in sex-specific analyses, covariate selection was intentionally limited to avoid model overfitting and unstable hazard ratio (HR) estimates. Variables included in the sex-specific analyses were age, race, area deprivation index (ADI) quartile, presence of atrial fibrillation, ATTR genotype, and NAC stage at diagnosis, based on clinical relevance.

### Statistical analysis

Statistical analyses were performed using R (version 4.5.1) and RStudio (version 2025.5.1.513). Baseline categorical variables were compared using Pearson's chi-square test or Fisher's exact test, as appropriate. Continuous variables were compared using the Wilcoxon rank-sum test and are presented as means ± standard deviations or medians with interquartile ranges. Differences in all-cause mortality were compared using Kaplan–Meier analyses with log-rank testing.

Multivariable Cox proportional hazards regression models were constructed to evaluate independent associations between clinical variables and all-cause mortality. The proportional hazards assumption for all Cox models was assessed using scaled Schoenfeld residuals, including both global and covariate-specific tests of proportionality. Restricted mean survival time (RMST) analysis was additionally performed at a prespecified truncation time of 60 months for analyses in which the proportional hazards assumption was questioned or when fewer than 50% of participants experienced the outcome of interest during follow-up. Firth-penalized Cox regression was performed as a sensitivity analysis to address potential small-sample and sparse-event bias. Additional multivariable and sex-stratified analyses were performed to evaluate potential confounding and sex-specific associations.

Variables with missing values were handled using complete-case analysis for multivariable models. We did not perform multiple imputation given the pattern and frequency of missingness. Statistical significance was determined by a *p*-value of <0.05.

Assessment of the proportional hazards assumption demonstrated no significant violation of the global proportional hazards assumption for the primary multivariable model (*χ*^2^ = 7.60, df = 11, *p* = 0.75). Similarly, no significant violations were observed for age at diagnosis (*p* = 0.922), atrial fibrillation (*p* = 0.863), amyloid stage (*p* = 0.143), race (*p* = 0.554), ADI (*p* = 0.677), or sex (*p* = 0.761). These findings suggest that the proportional hazards assumption was adequately met and that minor covariate-level deviations were unlikely to materially affect the primary conclusions.

## Results

### Baseline characteristics

The study included 334 patients with ATTR-CM, of which 268 (80%) were men and 66 (20%) were women (Table 1). Within the study population, female patients had lower pre-albumin levels at the time of diagnosis (19.9 mg/dL vs. 22.2 mg/dL; *p* = 0.041), a lower eGFR (49.4 mL/min/1.73m^2^ vs. 55.3 mL/min/1.73 m^2^; *p* = 0.002), higher log-transformed NT-proBNP levels (7.37 vs. 7.02; *p* = 0.033), a higher prevalence of the ATTR variant genotype than the wild-type (WT 56.1% vs. 84.3%, V 43.9% vs. 15.7%; *p* < 0.001), and were more likely to be African American (63.6% vs. 20.1%, *p* = 0.0001). There was no difference in mean age at diagnosis, presence of arrhythmias (atrial fibrillation, NSVT, or VT), or type of heart failure. The median follow-up time was 42 months from diagnosis.

**Table 1:** Baseline characteristics between female and male patients with ATTR-CM.

Variable	F	M	*p*-value
	*n* = 66	*n* = 268	
Age at diagnosis	79 ± 8	78 ± 9	0.5
Follow-up time, months	42 ± 31	38 ± 31	0.268
Race			**<0**.**001**
White (ref)	22 (33%)	200 (75%)	
African American	42 (64%)	54 (20%)	
Other	2 (30%)	14 (5.2%)	
Area deprivation index, quartile			**<0**.**001**
Q1 (ref)	6 (9.1%)	77 (28.7%)	
Q2	14 (21.2%)	69 (25.7%)	
Q3	17 (25.8%)	66 (24.6%)	
Q4, lowest ADI	28 (42.4%)	55 (20.5%)	
Atrial fibrillation	44/66 (66.7%)	201/267 (75.3%)	0.155
NSVT	48/66 (72.7%)	175/267 (65.5%)	0.266
Sustained VT	3/66 (4.5%)	11/267 (4.1%)	1.000
Genetic variant			**<0**.**001**
Genetic variant	29 (43.9%)	42 (15.7%)	
Wild-type	37 (56.1%)	226 (84.3%)	
Heart failure			0.780
No HF	4 (6.1%)	23 (8.6%)	
HFpEF	41 (62.1%)	159 (59.3%)	
HFrEF	16 (24.2%)	76 (28.4%)	
eGFR (mL/min/1.73 m^2^)	49 ± 16.8	55.3 ± 15.7	**0**.**002**
NT-proBNP, log-transformed^a^	7.37 ± 1.06	7.02 ± 1.10	**0**.**033**
Pre-albumin (mg/dL)	19.9 ± 7.7	22.2 ± 6.8	**0**.**041**
NAC stage			**0**.**002**
NAC stage 1	28 (50.0%)	170 (70.8%)	
NAC stage 2	16 (28.6%)	52 (21.7%)	
NAC stage 3	12 (21.4%)	0 (0%)	
NAC stage 4	18 (7.5%)	0 (0%)	

ADI, area deprivation index; ATTR, transthyretin amyloidosis; BMI, body mass index; eGFR, estimated glomerular filtration rate; HFpEF, heart failure with preserved ejection fraction; HFrEF, heart failure with reduced ejection fraction; NAC, National Amyloidosis Center; NSVT, non-sustained ventricular tachycardia; NT-proBNP, N-terminal pro-B-type natriuretic peptide; VT, ventricular tachycardia; WT, wild-type.

Continuous variables are presented as mean ± SD; comparisons were performed using the Wilcoxon rank-sum test. Categorical variables are presented as *n* (%); comparisons were performed using the chi-square or Fisher’s exact test (Fisher’s test when any expected cell count was <5). Bold *p*-values indicate statistical significance at *p* < 0.05.

a NT-proBNP was log-transformed (natural log) prior to analysis. Values are reported on the log scale as mean ± SD.

### Outcomes

#### NAC staging

Survival increased in a stepwise fashion with increasing NAC stage, with patients at NAC stage 1 experiencing the greatest survival (Figure 1). Female patients had a higher NAC stage than male patients at the time of diagnosis (Figure 2). The interaction between NAC stage and sex was evaluated using a Cox proportional hazards model, with stage 1 as the reference. Sex and amyloid stage were not statistically significant, indicating that a similar risk of mortality was consistent across NAC stages [sex (M) × stage 2: *p* = 0.832; sex (M) × stage 3: *p* = 0.261]. A likelihood ratio test was also performed for the interaction between sex and NAC stage, which demonstrated no significant interaction [*χ*^2^ = 3.52(M), df = 2, *p* = 0.172]. The individual interaction terms were non-significant (male × stage 2: HR 1.09, CI 0.23–5.21, *p* = 0.911; male × stage 3: HR 3.45, CI 0.73–16.39, *p* = 0.119). This finding suggests that stage mortality was homogeneous between male and female patients, although there were only 30 NAC stage 3 patients in the cohort.

**Figure 1:**
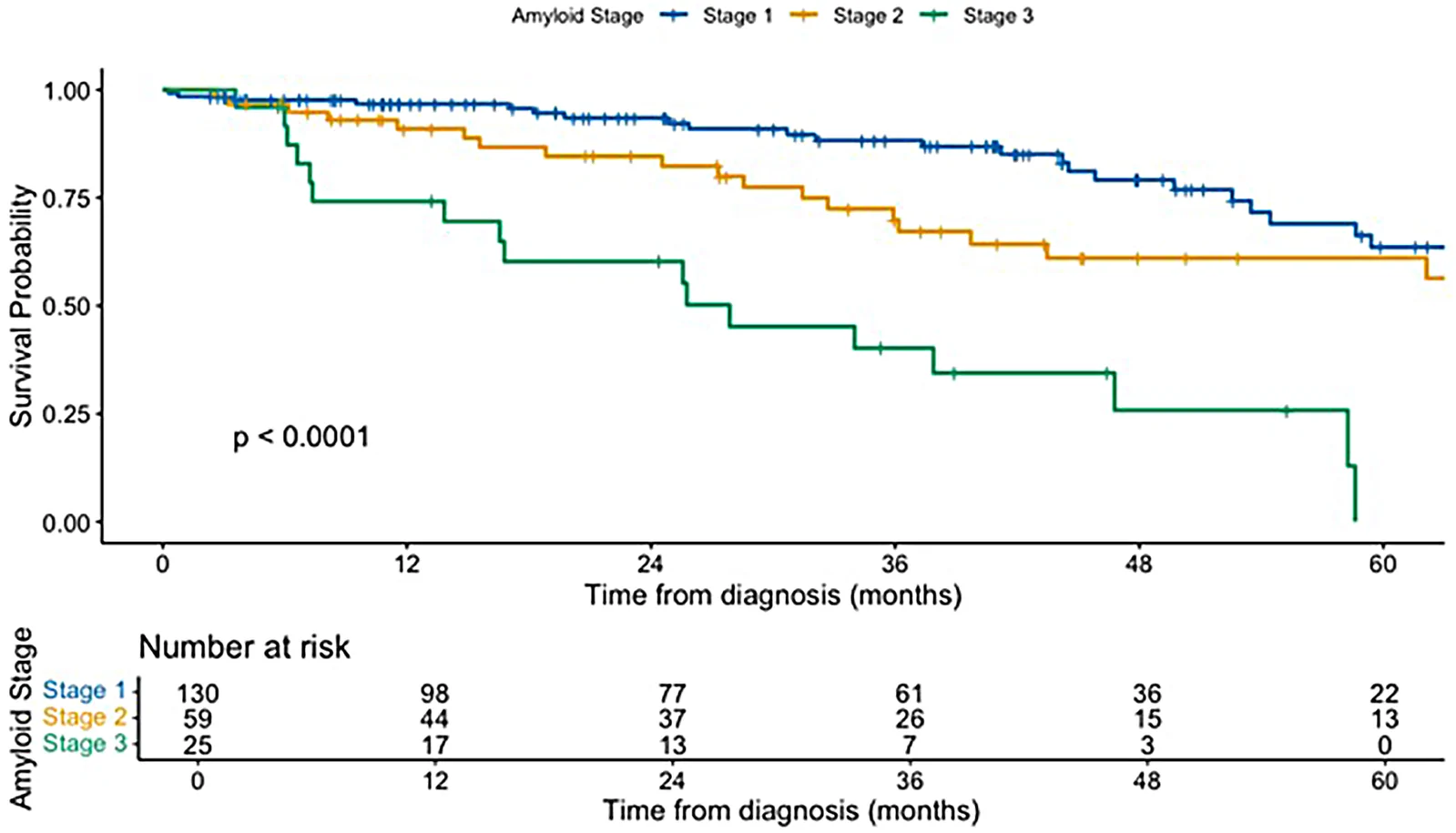
Kaplan–Meier estimates of all-cause mortality stratified by NAC stage. The solid lines represent Kaplan–Meier survival estimates of all-cause mortality stratified by NAC stage 1 (blue line), NAC stage 2 (yellow line), and NAC stage 3 (green line) at the time of diagnosis. Tick marks indicate censored data. A log-rank test was used to compare survival distributions; stage 2 vs. stage 1: HR = 3.05, CI = 1.33–7.01, *p* = 0.009; stage 3 vs. stage 1: HR = 7.17, CI = 3.22–16, *p* ≤ 0.001. The number of patients at risk at each time point is provided on the horizontal axis at the bottom of the figure.

**Figure 2:**
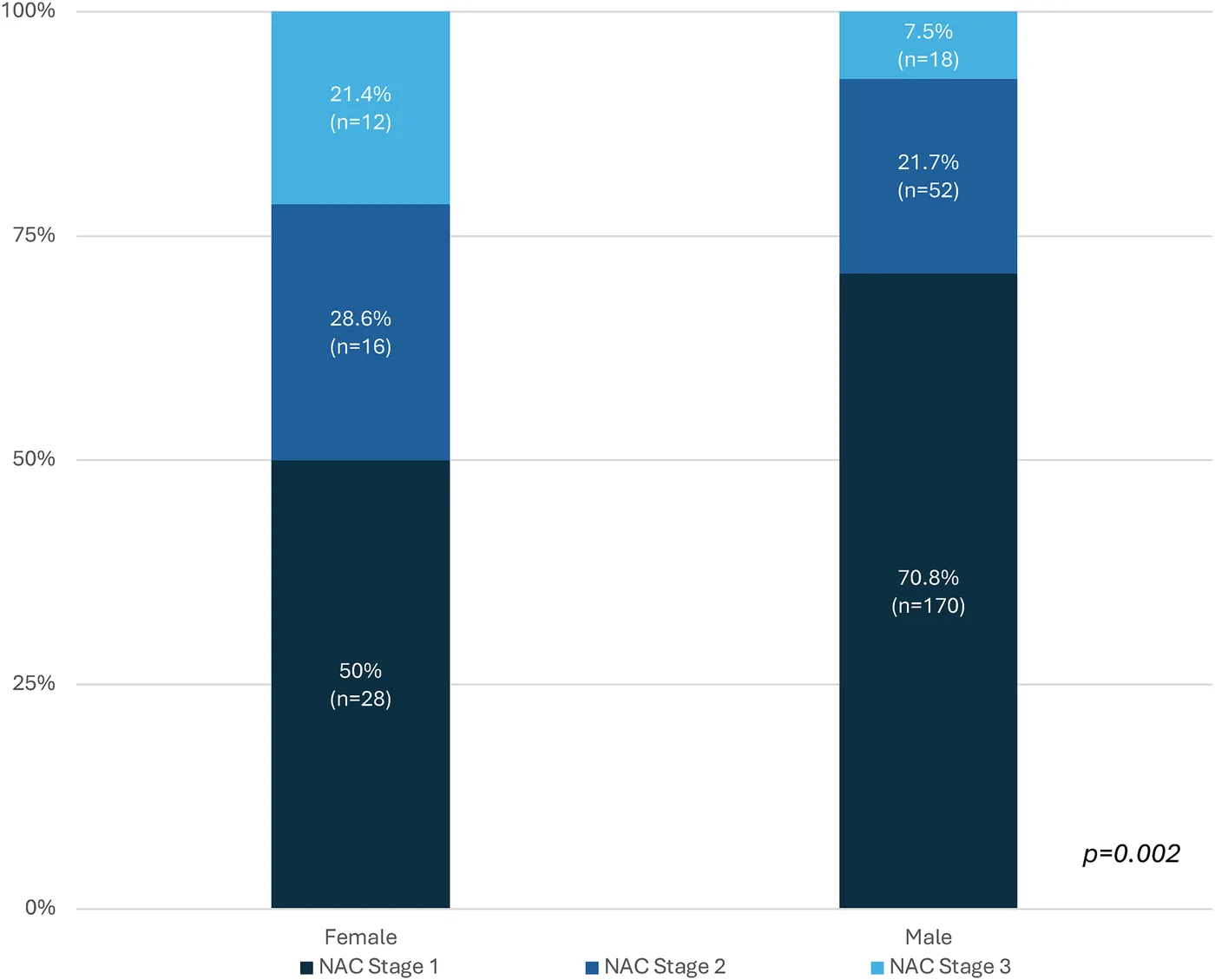
Distribution of NAC stage at diagnosis by sex. The stacked bar graph represents the NAC stage distribution between male and female patients. Female patients were diagnosed at higher NAC stages compared with male patients (stage 1: 50% vs. 71%; stage 2: 28.6% vs. 21.7%; and stage 3: 21.4% vs. 7.5%; *p* = 0.002).

### Survival

In the univariate analysis for survival, older age at diagnosis (HR 1.10, CI 1.03–1.14, *p* < 0.001), atrial fibrillation (HR 3.53, CI 1.62–7.66, *p* = 0.001), higher NT-proBNP levels (HR 1.70, CI 1.36–2.13, *p* < 0.001), and NAC stage 3 (HR 5.07, CI 2.84–9.07, *p* < 0.001) were associated with increased mortality (Table 2). African American race (HR 0.46, CI 0.26–0.84, *p* = 0.012), genetic variant (HR 0.37, CI 0.19–0.71, *p* = 0.003), and a higher eGFR (HR 0.96, CI 0.94–0.98, *p* < 0.001) were associated with decreased mortality (Table 2). In the multivariable analysis for mortality, age at diagnosis (HR 1.07, CI 1.03–1.11, *p* < 0.001), fourth-quartile area deprivation index (HR 2.19, CI 1.27–8.63, *p* = 0.040), atrial fibrillation (HR 3.31, CI 1.27–8.63, *p* = 0.014), and stage 3 NAC at diagnosis (HR 4.59, CI 2.39–8.80, *p* < 0.001) were associated with increased mortality (Table 3). Sex remained a non-significant factor (HR 1.25, CI 0.61–2.55, *p* = 0.545), along with race (Black HR 0.48, CI 0.22–1.04, *p* = 0.063) and variant status (HR 0.74, CI 0.30–1.83, *p* = 0.516) (Table 3).

**Table 2:** Univariable Cox regression analysis of mortality predictors.

Variable	HR	95% CI	*p*-value
Age at diagnosis	**1** **.** **10**	**1.06** **–** **1.14**	**<0** **.** **001**
Male sex (female ref)	1.17	0.65–2.08	0.600
Race			
White (ref)	—	—	—
African American (White ref)	0.46	0.26–0.84	0.012
Other	0.33	0.05–2.35	0.266
Area deprivation index, quartile			
Q1 (ref)	—	—	—
Q2	1.15	0.62–2.11	0.677
Q3	0.90	0.48–1.70	0.752
Q4, lowest ADI	1.30	0.71–2.38	0.402
Atrial fibrillation	**3** **.** **53**	**1.62** **–** **7.66**	**0** **.** **001**
NSVT	1.00	0.63–1.60	0.992
Sustained VT	0.36	0.09–1.46	0.152
Genetic variant (WT ref)	**0** **.** **37**	**0.19** **–** **0.71**	**0** **.** **003**
Heart failure			
No HF (ref)	—	—	—
HFpEF	2.24	0.72–7.64	0.158
HFrEF	1.51	0.44–5.14	0.510
eGFR	**0** **.** **96**	**0.94** **–** **0.98**	**<0** **.** **001**
NT-proBNP	**1** **.** **70**	**1.36** **–** **2.13**	**<0** **.** **001**
Pre-albumin	0.97	0.93–1.02	0.274
NAC stage			
NAC stage 1 (ref)	—	—	—
NAC stage 2	1.62	0.94–2.78	0.080
NAC stage 3	**5** **.** **07**	**2.84** **–** **9.07**	**<0** **.** **001**

ADI, area deprivation index; eGFR, estimated glomerular filtration rate; HFpEF, heart failure with preserved ejection fraction; HFrEF, heart failure with reduced ejection fraction; HR, hazard ratio; NAC, National Amyloidosis Center; NSVT, non-sustained ventricular tachycardia; NT-proBNP, N-terminal pro-B-type natriuretic peptide; VT, ventricular tachycardia; WT, wild-type.

Values are HR (95% CI). Bold indicates statistical significance at *p* < 0.05.

**Table 3:** Cox proportional hazards analysis for mortality.

Variable	HR	95% CI	*p*-value
Age at diagnosis	**1**.**07**	**1.03**–**1.11**	**<0**.**001**
Sex			
Female (ref)	—	—	—
Male	1.25	0.61–2.55	0.545
Race			
White (ref)	—	—	—
Black/African American	0.48	0.22–1.04	0.063
Other	0.34	0.015–2.49	0.286
Area deprivation index, quartile			
Q1 (ref)	—	—	—
Q2	1.11	0.91–3.20	0.096
Q3	1.24	0.61–2.51	0.550
Q4, lowest ADI	**2**.**19**	**1.04**–**4.64**	**0**.**040**
Atrial fibrillation	**3**.**31**	**1.27**–**8.63**	**0**.**014**
ATTR genotype			
Wild-type (ref)	—	—	—
Genetic variant	0.74	0.30–1.83	0.516
NAC stage			
NAC stage 1 (ref)	—	—	—
NAC stage 2	1.61	0.92–2.82	0.093
NAC stage 3	**4**.**59**	**2.39**–**8.80**	**<0**.**001**

ADI, area deprivation index; ATTR, transthyretin amyloidosis; CI, confidence interval; HR, hazard ratio; NAC, National Amyloidosis Center; PH, proportional hazards; WT, wild-type.

Values are HR (95% CI). Bold indicates statistical significance at *p* < 0.05. The NAC stage was missing in 40 patients (12%); ADI was missing in 4 additional patients. Core covariates and ADI (*N* = 293, events = 75).

#### Sex and mortality in ATTR-CM patients

The median follow-up time across the cohort was 42 months (IQR 17–70). In our analysis, female sex was not associated with poorer long-term survival compared with male patients in the Cox proportional hazards analysis (HR = 1.29; 95% CI 0.68–2.47; *p* = 0.43) (Figure 3). For additional assessment using a prespecified 60-month time horizon, the unadjusted RMST difference between male and female patients was −2.2 months [male: 50.78 (CI 48.47–53.09) vs. female: 52.97 (CI 48.75–57.18); *p* = 0.37] (Figure 4), indicating that male patients had, on average, 2.2 months less event-free survival than female patients over 5 years, although the difference was not significant. To address the effect of the small sample size, a Firth-penalized Cox regression model demonstrated similar long-term results (HR 1.34, CI 0.71–2.74; *p* = 0.384). Cumulative incidence was slightly higher among female patients at 1 year (female 6.4% vs. male 5.3%) but diverged thereafter, with male patients having a higher cumulative incidence at 3 years (12.5% vs. 19.4%) and 5 years (22% vs. 32.9%). Taken together with the RMST results, these findings suggest a numerical trend toward increased mortality among male patients, although the difference was not statistically significant.

**Figure 3:**
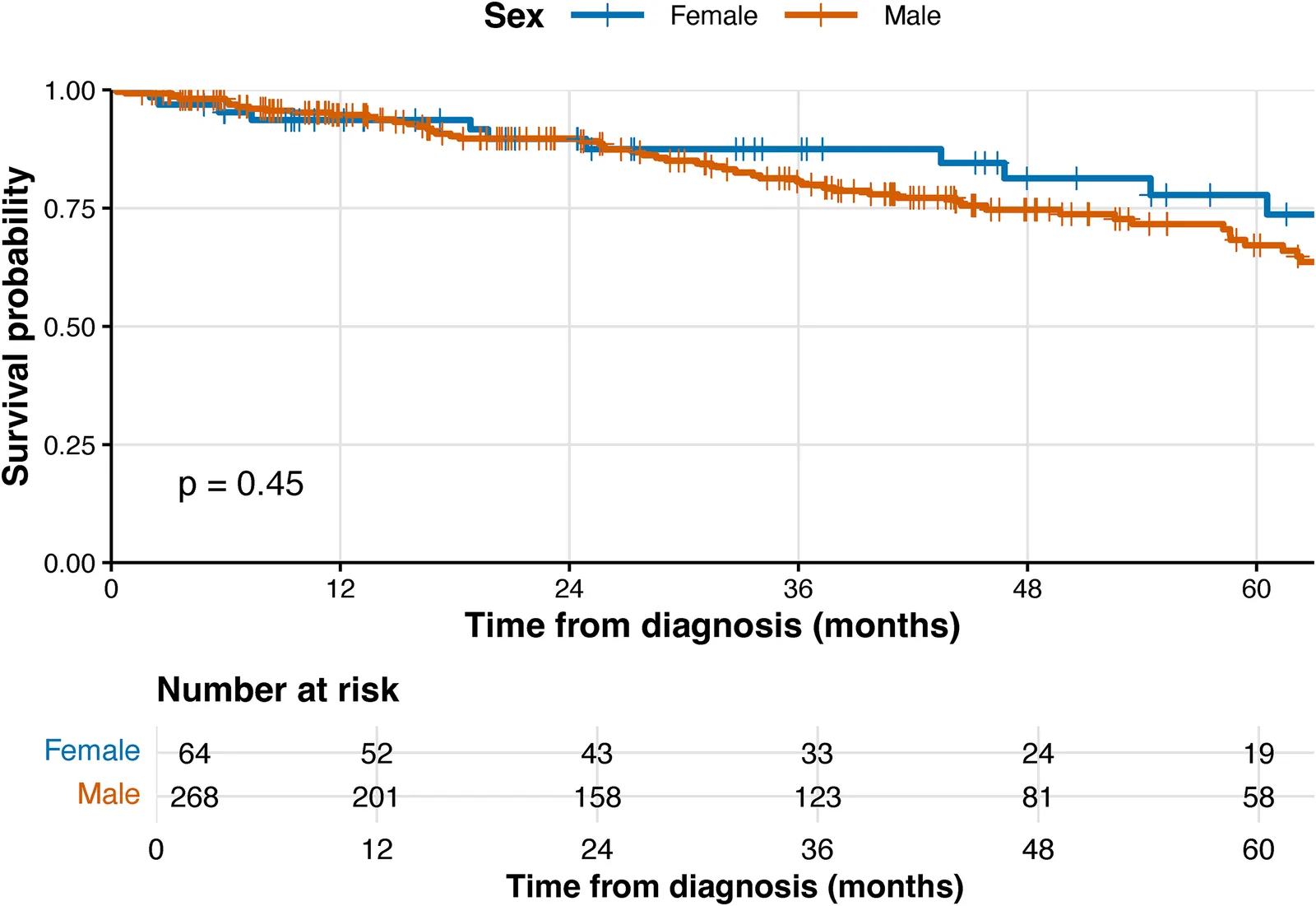
Kaplan–Meier estimates of all-cause mortality stratified by sex. The solid lines represent Kaplan–Meier survival estimates of all-cause mortality stratified by female (blue) and male (yellow) patients. Tick marks indicate censored data. A log-rank test was used to compare survival distributions (HR = 1.29, 95% CI 0.68–2.47, *p* = 0.43)*.* The number of patients at risk at each time point is provided on the horizontal axis at the bottom of the figure.

**Figure 4:**
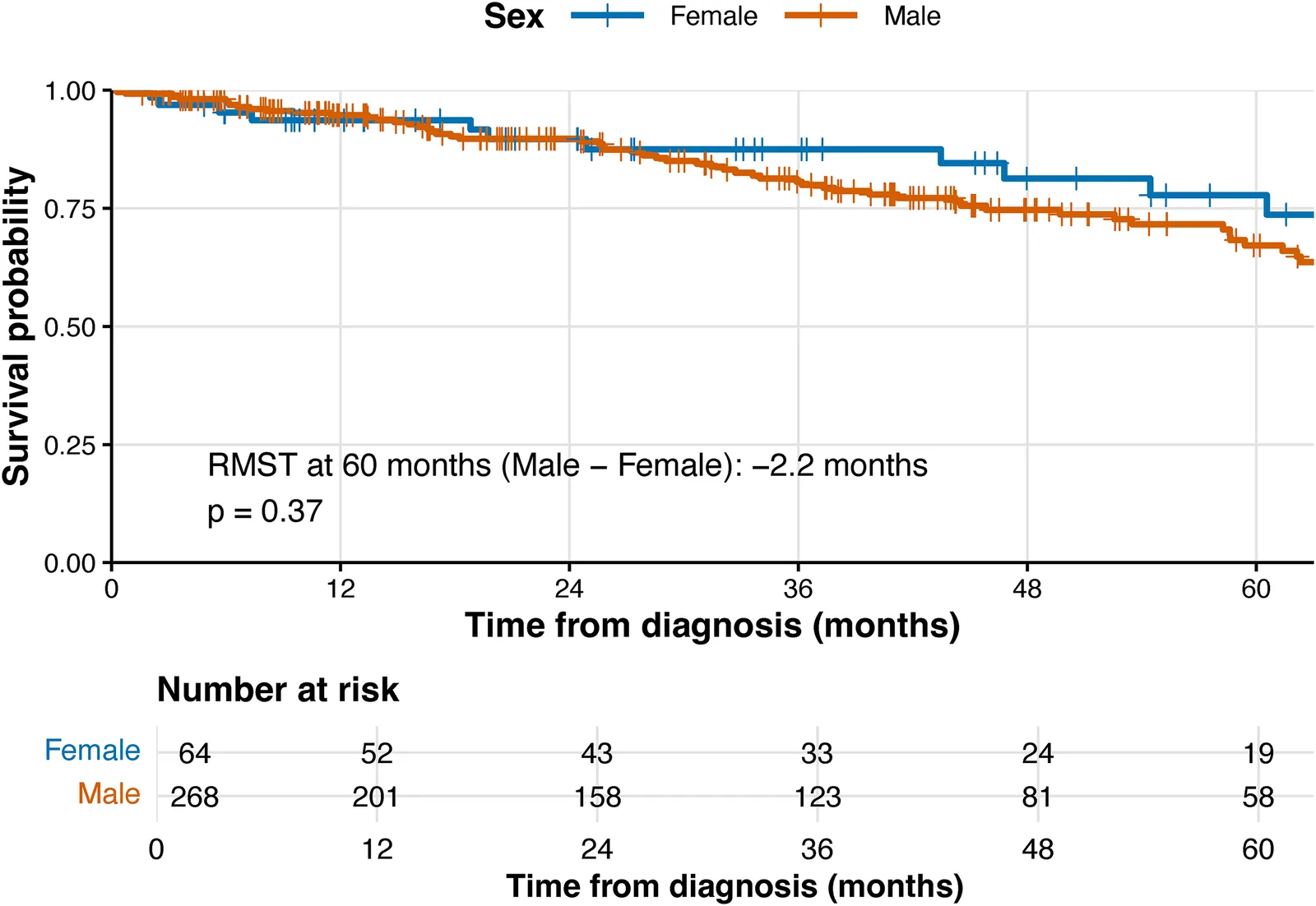
Five-year restricted mean survival time stratified by sex. the solid lines represent RMST of all-cause mortality stratified by female (blue) and male (yellow) patients. RMST was calculated using a truncation time of 60 months. Tick marks represent censored data. At 60 months, the RMST was 50.78 months (CI 48.47–53.09) for male patients and 52.97 months (CI 48.75–57.18) for female patients (*p* = 0.37), resulting in an absolute difference of 2.2 months. The number of patients at risk at each time point is provided on the horizontal axis at the bottom of the figure.

#### Hospitalization

In the univariate analysis for any hospitalization within the first year of diagnosis, age at diagnosis (HR 1.05, CI 1.02–1.08, *p* < 0.001), atrial fibrillation (HR 3.06, CI 1.85–5.97, *p* < 0.001), heart failure with preserved ejection fraction (HFpEF) (HR 4.89, CI 2.02–11.85, *p* < 0.001), heart failure with reduced ejection fraction (HFrEF) (HR 4.02, CI 1.58–10.25, *p* = 0.004) NT-proBNP (HR 1.25, CI 1.00–1.57, *p* = 0.047), and NAC stages 2 and 3 (stage 2: HR 2.67, CI 1.39–5.13, *p* = 0.003; stage 3: HR 2.77, CI 1.08–7.08, *p* = 0.033) were associated with all-cause hospitalization within the first year after diagnosis (Table 4). Other race (HR 0.34, CI 0.12–0.99, *p* = 0.047), eGFR (HR 0.96, CI 0.94–0.98, *p* < 0.001), and pre-albumin (HR 0.94, CI 0.89–0.98, *p* = 0.005) were associated with no hospitalizations within the first year of diagnosis (Table 4). Sex was not associated with hospitalization in the first year (male vs. female: HR 0.68, CI 0.38–1.22, *p* = 0.195).

**Table 4:** Univariable Cox regression analysis of first year all-cause hospitalizations.

Variable	HR	95% CI	*p-*value
Age at diagnosis	**1**.**05**	**1.02**–**1.08**	**<0**.**001**
Male sex (female ref)	0.68	0.38–1.22	0.195
Race			
White (ref)	—	—	—
African American (White ref)	0.88	0.54–1.46	0.628
Other	0.34	0.12–0.99	0.047
Area deprivation index, quartile			
Q1 (ref)	—	—	—
Q2	0.76	0.40–1.46	0.410
Q3	0.70	1.36–1.33	0.272
Q4, lowest ADI	0.67	0.35–1.28	0.230
Atrial fibrillation	**3**.**06**	**1.85**–**5.07**	**<0**.**001**
NSVT	1.52	0.95–2.44	0.082
Sustained VT	3.46	0.76–15.74	0.108
Genetic variant (WT ref)	0.61	0.36–1.05	0.072
Heart failure			
No HF (ref)	—	—	—
HFpEF	4.89	2.02–11.85	<0.001
HFrEF	4.02	1.58–10.25	0.004
eGFR	**0**.**96**	**0.94**–**0.98**	**<0**.**001**
NT-proBNP	**1**.**25**	**1.00**–**1.57**	**0**.**047**
Pre-albumin	0.94	0.89–0.98	0.005
NAC stage			
NAC stage 1 (ref)	—	—	—
NAC stage 2	2.67	1.39–5.13	0.003
NAC stage 3	**2**.**77**	**1.08**–**7.08**	**0**.**033**

ADI, area deprivation index; eGFR, estimated glomerular filtration rate; HFpEF, heart failure with preserved ejection fraction; HFrEF, heart failure with reduced ejection fraction; HR, hazard ratio; NAC, National Amyloidosis Center; NSVT, non-sustained ventricular tachycardia; NT-proBNP, N-terminal pro-B-type natriuretic peptide; VT, ventricular tachycardia; WT, wild-type.

Values are HR (95% CI). Bold indicates statistical significance at *p* < 0.05.

In the multivariable linear regression for all-cause hospitalization, atrial fibrillation (HR 2.96, CI 1.63-5.40, *p* < 0.001) and NAC stage 2 (HR 2.08, CI 1.04-4.19, *p* = 0.0040) were associated with any hospitalization during the first year after ATTR-CM diagnosis (Table 5). Race other than white and African American (HR 0.25, CI 0.08-0.85, *p* = 0.026) was associated with no hospitalizations during the first year after ATTR-CM diagnosis. ADI, variant status, and race were not associated with the secondary outcome of interest (Table 5).

**Table 5:** Multivariable logistic regression for all-cause hospitalization within 1 year of diagnosis.

Variable	OR	95% CI	*p*-value
Age at diagnosis	1.02	0.98–1.05	0.359
Sex			
Female (ref)	—	—	—
Male	0.73	0.34–1.56	0.415
Race			
White (ref)	—	—	—
Black/African American	1.05	0.52–2.14	0.893
Other	0.25	0.08–0.85	0.026
Area deprivation index, quartile			
Q1 (ref)	—	—	—
Q2	0.65	0.30–1.39	0.265
Q3	0.61	0.28–1.33	0.214
Q4, lowest ADI	0.55	0.25–1.24	0.150
Atrial fibrillation	**2**.**96**	**1.63**–**5.40**	**<0**.**001**
ATTR genotype			
Wild-type (ref)	—	—	—
Genetic variant	0.88	0.40–1.94	0.757
NAC stage			
NAC stage 1 (ref)	—	—	—
NAC stage 2	**2**.**08**	**1.04**–**4.19**	**0**.**040**
NAC stage 3	2.18	0.77–6.17	0.142

ADI, area deprivation index; HFpEF, heart failure with preserved ejection fraction; HFrEF, heart failure with reduced ejection fraction; HR, hazard ratio; NAC, National Amyloidosis Center; WT, wild-type.

Values are HR (95% CI). Bold indicates statistical significance at *p* < 0.05.

## Discussion

In this contemporary cohort of 334 patients with ATTR-CM, we report the following findings: (1) women comprised 20% of the ATTR-CM population, (2) women were diagnosed at a later NAC stage, and (3) despite having a higher proportion of factors associated with increased mortality, women had survival probability similar to men.

In our study, we highlight the well-documented disparity in diagnosis, as only 20% of the study population were women. Although prior studies have shown a male predominance in both wild-type and variant forms of transthyretin amyloid cardiomyopathy, literature comparing active screening-based and referral-based ascertainment of women with ATTR-CM suggests that the prevalence of the disease among women is likely higher than we currently recognized (11, 17). In addition to differences in the proportion of men and women at diagnosis, we found that women are more likely to experience greater socioeconomic difficulties based on area deprivation, have a higher prevalence of genetic variants, have a worse biomarker profile, and be diagnosed at later NAC stages.

Shetty et al., in a study of determinants of transthyretin levels and their association with adverse clinical outcomes in the United Kingdom Biobank, included participants free of renal and cardiovascular disease and reported that transthyretin levels were lower in women and that lower levels were associated with all-cause mortality (18).

In a landmark study of survival in 869 patients with transthyretin cardiac amyloidosis, Gillmore et al. used the biomarkers NT-proBNP and estimated glomerular filtration rate to develop and validate the NAC staging system for transthyretin cardiac amyloidosis. An NT-proBNP level of <3,000 and an eGFR of >45 defined stage I and was associated with a median survival of 69 months. An NT-proBNP level of >3,000 and an eGFR of <45 defined stage III and was associated with a median survival of 24.1 months (4). Zampieri et al., in a study including 259 patients with wild-type transthyretin cardiac amyloidosis, found that women were diagnosed at later NAC stages but had no statistically significant difference in survival compared to men (12). Conversely, a large study involving 1,732 patients by Patel et al. found that women presented at a later NAC only in wild-type ATTR-CM compared with variant ATTR-CM and had similar survival across the entire cohort (13).

Shankar et al., in a study of 282 patients with transthyretin amyloid cardiomyopathy stratified by race and area deprivation, found that Black patients from areas of high deprivation had a significantly greater hazard of heart failure hospitalization or death than White patients, independent of disease stage at the time of diagnosis (19). Miller et al. reported that disparities associated with socioeconomic status were associated with treatment delays (20).

Despite having a higher risk profile for mortality, female patients with transthyretin amyloidosis had survival outcomes similar to male patients with transthyretin amyloidosis. The mechanisms underlying later-stage presentation in female patients remain incompletely understood, although current literature has begun to explore why female patients are diagnosed at later stages. Leading hypotheses include systemic under-recognition, atypical symptom presentation, and smaller cardiac anatomy as contributing factors, with the latter being particularly relevant when left ventricular hypertrophy is not indexed to body size (7, 8, 11, 13). These diagnostic barriers may lead to delayed referral and evaluation, resulting in a later stage at diagnosis.

An additional consideration is whether the NAC staging system itself accurately reflects disease severity in female patients. NAC is a composite of eGFR and NT-proBNP, and while eGFR uses a sex-based correction (21), NT-proBNP does not, despite studies demonstrating female patients have higher NT-proBNP levels independent of cardiac disease (22, 23). If female patients are staged at NAC 2 or 3 with lower infiltration, this could reflect an overestimation that partially explains the similarity in survival compared with men. Future studies should investigate sex-specific NT-proBNP cutoffs and examine the relationship between NAC stage and degree of myocardial infiltration between male and female patients.

Finally, these findings also raise questions about protective biological factors in female patients that mitigate advanced disease state and socioeconomic factors associated with this disease. Mechanisms worth investigating include differences in sex-based amyloid fibril type and the impact of estrogen on TTR synthesis and infiltration (14, 24). Socioeconomic factors related to delays in diagnosis or access to specialized treatment centers also remain an important area of investigation.

### Limitations

There are several limitations when interpreting the findings from this study. First, this was a single-center retrospective study of individuals referred to a quaternary hospital, which limits generalizability. In addition, death data were obtained through chart review, and the cause of death could not be obtained to specifically stratify cardiac death. Information was also lacking regarding the ATTR disease-specific medications that individual patients may have received and the duration of treatment, which could have affected survival and thus introduced confounding. Finally, there were fewer primary outcome events in the subgroup analysis, which may mask a signal of difference when comparing male and female patients by NAC stage. Despite these limitations, the results are similar to those of studies conducted before and after the advent of ATTR-directed therapeutics and add to the body of literature on sex differences in transthyretin cardiac amyloidosis.

## Conclusion

In our study assessing gender differences in ATTR-CM diagnosis and long-term outcomes, female patients with ATTR-CM were diagnosed at later NAC stages and had more risk factors associated with mortality. Despite these disparities, female patients with ATTR-CM had long-term outcomes similar to male patients. Our findings may suggest that NAC stage overestimates disease severity in female patients or, alternatively, that biological mechanisms confer a less aggressive disease phenotype in female patients despite advanced disease staging.

## Data Availability

The original contributions presented in the study are included in the article/Supplementary Material; further inquiries can be directed to the corresponding author.
